# Symbiotic Bacterial Communities of Insects Feeding on the Same Plant Lineage: Distinct Composition but Congruent Function

**DOI:** 10.3390/insects15030187

**Published:** 2024-03-12

**Authors:** Waleed Afzal Naveed, Qian Liu, Congcong Lu, Xiaolei Huang

**Affiliations:** Key Laboratory of Ecological Pest Control for Fujian and Taiwan Crops, College of Plant Protection, Fujian Agriculture and Forestry University, Fuzhou 350002, China; waleedafzal75@gmail.com (W.A.N.); liuqian9502@163.com (Q.L.); lcchuaer613@126.com (C.L.)

**Keywords:** bamboo insects, diet, phylogeny, bacterial symbionts, 16S sequencing

## Abstract

**Simple Summary:**

This research sheds light on the intricate connections between *Bambusa*-feeding insects and their symbiotic bacteria. It represents the first comparative analysis of bacterial communities across insects from three distinct orders, all feeding on the same *Bambusa* species. The findings underscore substantial variations in symbiotic bacteria across samples, influenced by dietary choices, seasonal variations, and host phylogeny. While insects feeding on the same plant lineage exhibit different bacterial communities, their core functional abilities remain similar. These insights contribute to our understanding of insect–bacterial interactions, providing implications for insect biology and ecology.

**Abstract:**

The health and diversity of plant-feeding insects are strictly linked to their host plants and mutualistic symbionts. However, the study of bacterial symbionts within different insects on the same plant lineage is very limited. This study aimed to investigate the bacterial diversity in insect samples that exclusively feed on *Bambusa*, representing three insect orders, Hemiptera, Lepidoptera, and Blattodea, each exhibiting distinct dietary preferences. The bacterial community was predominantly composed of *Proteobacteria*, *Spirochaetota*, *Cyanobacteria*, *Firmicutes*, and *Bacteroidota*. The study found significant variations in symbiotic organisms among three insect orders: hemipterans had *Buchnera*, lepidopterans had *Acinetobacter*, and blattodean had *Treponema*. Furthermore, the dietary preferences of these insects played a pivotal role in shaping the symbiotic relationship of insects. *Proteobacteria* are prevalent in sap feeders, *Spirochaetota* dominate in stem feeders, and *Cyanobacteria* are abundant in leaf feeders. Seasonal influences also affect bacterial symbionts in *P. bambucicola*, with *Serratia* present exclusively in winter. We also observed that the bacterial composition varies across all samples, but their core functions appear to be consistent. This highlights the complex relationship between host phylogeny and diet, with phylogeny being the primary driver, shaping adaptations to specialized diets.

## 1. Introduction

Insects are known for their ubiquity and adaptability across diverse environments [1] and are referred to as “polyorganisms” due to their coexistence with a diverse microbiota that resides within their bodies or tissues [2]. These microbes include bacteria, fungi, viruses, protozoa, and archaea contributing to various physiological aspects including immunity, metabolism, and nutrition [2,3,4]. Insect-associated bacterial symbionts exert diverse evolutionary and ecological influences on hosts [3,5], for example, *Wolbachia*, identified as the primary modulator of host biology in insects [6]. Meanwhile, *Buchnera* and *Wigglesworthia* serve as vital nutrient suppliers to aphids and tsetse flies, respectively [7].

The interactions between bacteria and insects are common in the natural world. Furthermore, recent studies have discovered the significant influence of symbiotic bacteria on the behavior of insects. For instance, symbionts influence the egg-laying behavior of *Drosophila melanogaster* [8], host-plant specialization in aphids [4], crawling behavior between nymphs of stink bugs [9], and foraging behavior in fruit flies [10]. Symbionts influence these behaviors mainly through changes in metabolism, dietary habits, and sensory responses [8]. The diversity of symbionts in insects can be attributed to different factors such as host species [11], host-plant specialization [12], and climate [12,13], and the same insect species might harbor distinct symbiotic communities based on varying feeding habitats [14] and different times [15]. Additionally, symbiont diversity and prevalence frequently vary among host species and populations [13].

Insect-associated microbes, particularly those in higher abundance, are more likely to play a pivotal role in host adaptation. Host phylogeny and diet are recognized as two primary factors that play a significant role in shaping the composition and function of animal symbionts [16]. In addition, the diet and living environment of insects may result in differences in bacterial communities and dynamics [17]. Diet can potentially impact symbionts [2]. For instance, isogenic *Drosophila melanogaster* fed various diets exhibit diverse bacterial communities, while three genetically distant Drosophilids fed the same diet display similar bacterial microbiomes [14]. Relying significantly on coevolution with bacterial symbionts has notable consequences in animals, particularly leading to the occurrence of dietary specialization. This phenomenon represents a crucial convergent evolutionary event, enabling them to adapt to a variety of dietary habitats [18]. This specialization is particularly pronounced in phytophagous insects, where the absence of endogenous celluloses necessitates the mediation or provision of cellulose digestion by symbiotic bacterial communities [19]. Notably, as hosts, animals also exert influence on microbes in terms of morphology, physiology, and ecology, taking part in the convergent evolution of the bacterial community [20].

Within the natural ecosystem, herbivores have evolved remarkable digestive systems that interact closely with microbial communities [21]. An analysis of the natural ecosystems where bamboo decomposition occurs has shown that herbivores have particularly good digestive systems [21,22]. Herbivores have benefited from interactions with bacterial communities selected by evolutionary processes over thousands of years to digest biomass [21,23]. Bamboo, a colossal tree-like grass, stands as one of the most crucial biomass resources globally [24]. Bamboo comprises cellulose fibers, which are nature’s fundamental building blocks for living organisms, embedded within a lignin matrix. The fibers align longitudinally within bamboo, providing exceptional tensile strength, flexural strength, and rigidity in a specific direction. Cellulose is typically found in association with other components such as hemicellulose, lignin, cutin, silica, and protein within cell walls [25]. As a result, cellulose effectively functions as a comprehensive defense mechanism [26]. Researchers highlight the fact that the majority of herbivorous insects lack the capacity to break down cellulose [26]. Bamboo-feeding insects symbolize a great example of mutualism [27]. Based on their feeding habits, bamboo-feeding insects can be classified into three categories, leaf, shoots, and sap feeders, in alignment with the bamboo’s structural components [28]. Additionally, it is widely accepted that dietary nutrition has a substantial influence on animal symbionts [29]. Over millennia, these herbivores have coevolved with symbiotic bacterial partners, shaping their capacity to digest biomass [21].

*Bambusa*, being a diverse genus of clumping bamboos thriving in subtropical regions, serves as a rich habitat attracting a wide array of insects from various orders. This diversity presents a valuable opportunity to investigate a hypothesis: insects feeding on the same plant lineage may share similar dominant symbiotic bacteria, or have distinct symbiotic bacterial compositions with similar functions, to adapt to that plant lineage. Considering that the symbiotic bacterial communities present in insects that feed on *Bambusa* could be determined by both the phylogeny of the host insects and their dietary habits, it is more plausible to anticipate varying symbiotic bacterial compositions among different insect species, but these bacterial communities have similar predicted functions due to feeding on the same plant groups.

In this study, we explored the bacterial communities residing within *Bambusa*-feeding insects by using 16S rRNA gene amplicon sequencing. We also investigated the stability of symbiotic bacterial composition over time, considering variations across different seasons and potential influencing factors. Our research endeavors to uncover the involved relationships among *Bambusa*-feeding insects and their bacterial partners, shedding light on whether shared host plants or insect phylogeny are the driving forces shaping these crucial associations.

## 2. Materials and Methods

### 2.1. Sample Collection

In 2022–2023, we conducted extensive insect collection exclusively from *Bambusa*-rich regions in Fuzhou, China. Our samples encompassed a diverse array of species, including *Oligia apameoides*, *Reticulitermes flaviceps*, *Discophora sondaica*, *Pseudoregma bambucicola*, *Ceratoglyphina styracicola*, *Takecallis taiwana*, *Ceratovacuna keduensis*, *Antonina pretiosa*, *Purohita taiwanensis*, and *Tropidocephal brunnipenni*. These specimens were thoughtfully chosen to provide a comprehensive representation of *Bambusa*-feeding insects. The detailed sample information used in our study is described in Table 1.

### 2.2. DNA Extraction and Validation

For DNA extraction, we used 2–3 individuals for small-bodied insects, while larger insect samples required only 1 individual. All samples underwent a thorough washing process, involving three washes with ultra-pure water. Subsequently, we conducted total genomic DNA extraction using the DNeasy Blood and Tissue Kit from QIAGEN (Hilden, Germany). To avoid environmental DNA contamination, DNA extraction was performed on an ultra-clean workbench. The bacterial universal primers 8F (5′-AGAGTTTGATCCTGGCTCAG-3′) and 1492R (5′-GGTTACCTTGTTACGACTT-3′) [30] were used for a PCR amplification process to verify the success of the DNA extraction. To further guarantee the accuracy of results, sterile deionized water served as a negative control. PCR amplification was conducted using a 25 µL reaction mixture, which included 1 µL of DNA, 2.5 µL of 10× LA PCR Buffer-II (Mg^2+^ added), 0.5 µL of dNTP mix (2.5 µL), 0.5 µL of each primer (10 µM), 0.5 µL of TaKaRa LA-Taq (5 µ/µL) from TaKaRa Bio Inc., Shiga, Japan, and 19.5 µL of water. ProFlexTM Base (Applied Biosystems, Inc., Waltham, MA, USA) was used for the amplification. The cycling conditions were as follows: 4 min of initial denaturation at 94 °C; 30 cycles of denaturation at 94 °C for 30 s; 40 s of annealing at 65 °C; 90 s of extension at 72 °C; and 10 min of final extension at 72 °C. The PCR results were visualized on a 1% agarose gel, and positive samples (approximately 1500 bp) were preserved at −20 °C for subsequent 16S library preparation. Notably, negative controls did not yield any bands, confirming the absence of contamination in our DNA extraction process.

### 2.3. 16S rRNA Gene Amplification and Sequencing

We focused on amplifying the V3–V4 hypervariable region of the 16S rRNA gene, spanning approximately 420 base pairs. To achieve this, we employed the following primers: 338F (5′-ACTCCTACGGGAGGCAGCA-3′) and 806R (5′-GGACTACHVGGGTWTCTAAT-3′) [31]. Our approach involved two distinct polymerase chain reaction (PCR) steps. The first PCR step targeted the specific gene regions of interest, while the second PCR step was dedicated to incorporating indices and adapter sequences. To assess the success of our PCR amplification, we conducted 1.8% agarose gel electrophoresis. After being purified and homogenized, the positive PCR results were used to build a sequencing library. This process allowed us to selectively amplify the desired 16S rRNA gene region and prepare the samples for high-throughput sequencing (HTS).

### 2.4. Sequencing Data Analysis

FLASH v1.2.11 was used to merge paired-end readings, requiring a minimum overlap of 10 base pairs. Subsequently, Trimmomatic v0.33 [32] was employed to further refine the combined raw tags, discarding any reads with a quality score less than 20 within a 50-base pair sliding window. Additionally, tags smaller than 300 base pairs were filtered out. To ensure the generation of high-quality, clean tags, chimera sequences were deleted from the dataset by UCHIME v8.1. Rarefaction normalization was employed to account for the unevenness in sequence depth across samples. Using USEARCH v10.0, the remaining sequences were classified into operational taxonomic units (OTUs) at a 97% similarity level. OTUs that made up less than 0.005% of all sequences were eliminated [33]. For further annotation, the sample sequences with the highest abundance within each OTU cluster were identified. Using the RDP classifier v2.2, taxonomic classifications were applied to all OTUs according to the SILVA database (Release 138.1). Additionally, BLAST searches against GenBank sequences were conducted to corroborate taxonomic designations.

### 2.5. Diversity and Statistical Analysis

To evaluate species richness and community diversity based on the operational taxonomic unit (OTU) table, we employed Mothur v1.30 [34] to calculate various diversity indices. These comprised the Shannon and Simpson indices for assessing community diversity and the Chao1 and ACE indices for estimating species richness. Larger values of Chao1, ACE, Shannon, and smaller values of Simpson indicate higher diversity within the sampled communities. Due to the different symbiotic bacterial compositions, samples were compared with the student *t*-test. To assess differences in symbiotic bacterial composition across various sample groups, we conducted beta diversity analysis. We used OTU-based Weighted Unifrac distances and Bray–Curtis distances for this purpose, which consider phylogenetic information associated with the OTUs. These distance metrics allowed us to measure community dissimilarity and estimate beta diversity, accounting for the quantity and presence of bacteria [35].

The nonmetric multidimensional scaling (NMDS) analysis of bacterial communities was conducted using the Vegan v2.3.0 package [36], and two-dimensional plots were generated with ggplot2 v3.1.1 [37] in the R v3.1.1 programming environment. NMDS is considered reliable when the stress value is less than 0.2. To assess statistically significant differences in symbiotic bacterial composition across all samples, three different insect orders (Hemiptera, Lepidoptera, Bllatodea), feeding habitat (Sap, Leaf, and Stem), and two seasons (summer and winter), we used Permutational Multivariate Analysis of Variance (PERMANOVA) and Analysis of Similarities (ANOSIM). These analyses were performed based on the Bray–Curtis distance and Weighted UniFrac distance matrices. ANOSIM and PERMANOVA were carried out using the Vegan v2.3.0 [36] package’s anosim and adonis functions, respectively, in the R programming environment version 3.1.1. In ANOSIM, an R-value closer to 1 suggests high dissimilarity between distinct groups compared to within groups [38]. A higher *R*^2^ value in PERMANOVA indicates that the grouping factor plays a more significant role in explaining overall variation [39]. High test reliability is indicated by *p*-values for both ANOSIM and PERMANOVA that are less than 0.05.

To explore potential functional variations in symbiotic bacterial communities, PICRUSt2 v2.3.0b0 [40] was employed to forecast the composition of the microbiome’s functional genes. This involved a comparison of bacterial species composition data derived from 16S rRNA gene sequences. The data generated by PICRUSt2 underwent analysis at the KEGG [41] orthology at level 3. We utilized STAMP v2.1.3 [42] to assess the significance of differences in the relative abundance and a *t*-test was conducted individually between the groups, with a significance threshold of *p* ≤ 0.05 indicating statistical significance.

## 3. Results

### 3.1. Analysis of 16S rDNA Sequencing

We acquired a total of 2,290,179 clean tags after quality control (an average of 69,399 tags per sample). Subsequent sequence filtering yielded 2,257,489 reads, with an average of 68,408 reads per sample (Table S1). The paired sequences of the 16S rRNA gene assemblies had an average length of 421 base pairs. These high-quality reads were classified into 731 OTUs (Table S2). For a comprehensive view of the dominant symbiotic bacteria taxa in all samples, please refer to Table S3, which provides the read distribution for the top 10 taxa.

### 3.2. OTUs Distribution and Comparison of Symbionts between Diets and Phylogeny

**Figure 1:**
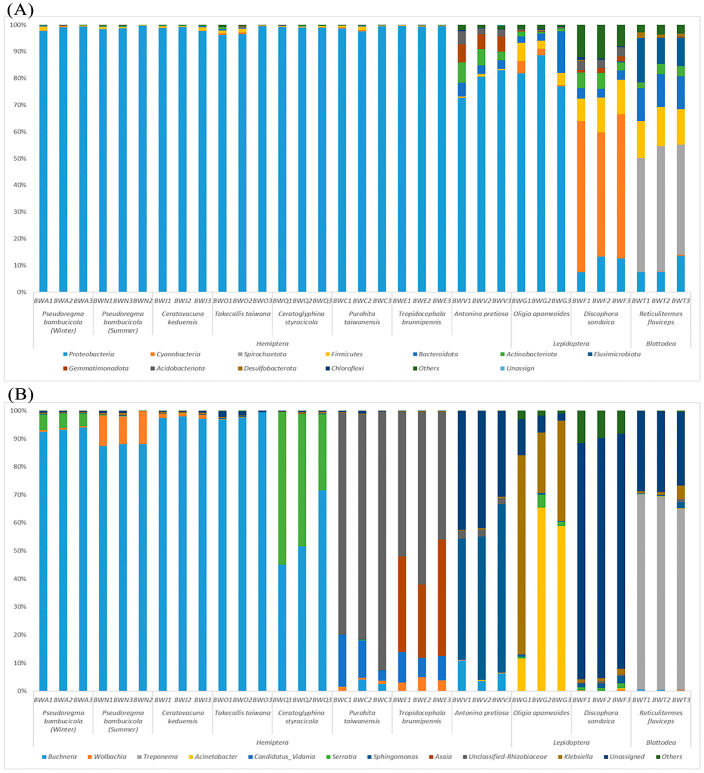
The taxonomic composition and proportional abundance of symbiotic bacteria linked to every sample (**A**) at the phylum level and (**B**) at the genus level.

**Figure 2:**
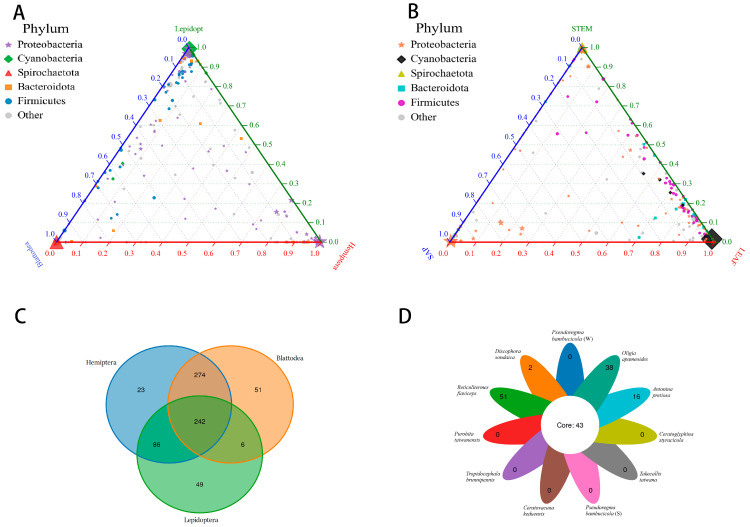
Plot showing the OTU distribution in ternary form. (**A**) Across three different insect orders. (**B**) Across three different diets. A single OTU is represented by each dot, and its size and location indicate how closely associated it is with certain insect groups and how abundant it is overall. (**C**) Venn diagram at OTU level across three different insect orders. (**D**) Flower plot between all groups. The top five enriched OTUs are represented by colored dots, while OTUs that are not significantly enriched in various insect orders are represented by gray dots.

The Shannon–Wiener curves, as depicted in Figure S1, eventually plateaued, indicating that our sequencing depth was adequate for subsequent data analysis. The bacterial community was categorized at various taxonomic levels, resulting in the classification of the 731 bacterial OTUs into 25 phyla, 45 classes, 109 orders, 190 families, and 331 genera. The relative distribution of major phyla is shown in Figure 1A. Sequences that could not be assigned to recognized bacterial phyla were labelled as “unassigned”, comprising 1.2% of the entire dataset. The category labeled ‘Others’ represents a small number of phyla (0.01% of the total sequences) sporadically present in certain samples in low-abundance patterns. At the phylum level, samples predominantly contained *Proteobacteria* (82.62% of total sequences), *Spirochaetota* (4.05%), *Cyanobacteria* (3.21%), and *Firmicutes* (2.64%). Notably, *Proteobacteria* were predominantly found across all species, particularly in hemipteran species with higher abundance (Figure 2A) and sap feeders (Figure 2B). *Spirochaetota* was mainly found in blattodean species as well as in stem feeders. While *Proteobacteria*, *Cyanobacteria*, and *Firmicutes* were mainly found in lepidopteran species (Figure 2A).

In our samples, a total of 331 bacterial genera were recognized, with the top 10 genera being *Buchnera*, Unclassified-Rhizobiaceae, *Serratia*, *Treponema*, *Sphingomonas*, *Asaia*, *Klebsiella*, *Acinetobacter*, *Candidatus_Vidania*, and *Wolbachia*. *Buchnera* was present in all collected aphid samples (Table S3). Unclassified-Rhizobiaceae was present in plant hoppers with higher abundance (Table S3). Lepidopteran insects exhibited a significant dominance of *Acinetobacter* (65%), a notable distinction from other sample types. Intriguingly, *Treponema* was exclusively detected in blattodean insects with high abundance (Figure 1B).

Among the 731 OTUs, maximum OTUs were found in *Reticulitermes flaviceps* followed by *Antonina pretiosa*, *Pseudoregma bambucicola* (summer), *Ceratovacuna keduensis*, *Ceratoglyphina styracicola*, *Discophora sondaica*, *Pseudoregma bambucicola* (winter), *Oligia apameoides*, *Purohita taiwanensis*, *Tropidocephala brunnipennis*, and *Takecallis taiwana* (Table S2). In total, 242 OTUs were shared among the three orders as shown in the Venn diagram, although 51, 49, and 23 OTUs were specific to Blattodea, Lepidoptera, and Hemiptera, respectively (Figure 2C). The most specialized OTUs were found in Blattodea. In contrast to the comparisons between the others, Hemipterac, and Blattodea shared more OTUs (Figure 2C). All the samples shared 43 common symbionts, which were present in all of them (Figure 2D, Table S4), while *Reticulitermes flaviceps* had maximum unique symbionts followed by *Oligia apameoides*, *Antonina pretiosa*, and *Discophora sondaica* (Figure 2D).

### 3.3. Alpha Diversity

**Figure 3:**
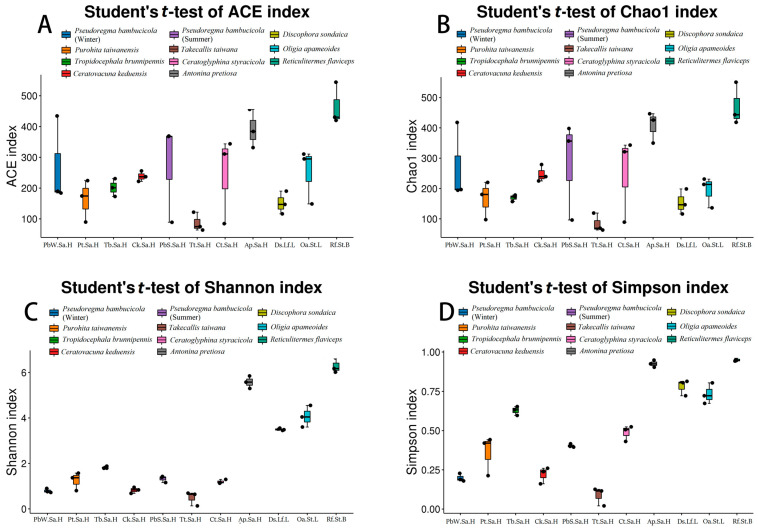
Comparison of the bacterial communities’ alpha diversity across all groups feeding on Bambusa, (**A**) ACE index, (**B**) Chao1 index, (**C**) Shannon index, and (**D**) Simpson index. Note: X-axis: Group name; Y-axis: Alpha diversity indices. The line inside the boxplots represents medians, the dots in the center are the means, and the whiskered bars are maximal and minimal values.

To evaluate the diversity of bacteria (Simpson and Shannon) and species richness (Chao1 and ACE), alpha diversity indices were utilized among all insects that feed on *Bambusa* (Table S2). The analysis revealed notable dissimilarities in bacterial communities among the various samples (Figure 1 and Table S2). All samples showed a broad distribution of alpha diversity indices for the symbiotic bacterial community, ranging from 63.82 to 544.25 (248.967 ± 21.89) for the ACE index, from 63.14 to 550.78 (245.16 ± 22.40) for the Chao1 index, from 0.13 to 6.63 (2.46 ± 0.34) for the Shannon index, and 0.02 to 0.96 (0.52 ± 0.05) for the Simpson index (Table S2, Figure 3C,D). The ace and chao1 indexes revealed that the richness of symbionts in *Reticulitermes flaviceps* was highest followed by *Antonina pretiosa*, *Pseudoregma bambucicola* (summer), *Ceratovacuna keduensis*, *Ceratoglyphina styracicola*, *Discophora sondaica*, *Pseudoregma bambucicola* (winter), *Oligia apameoides*, *Purohita taiwanensis*, *Tropidocephala brunnipennis*, and *Takecallis taiwana*, which exhibited the lowest richness levels. The values of all the samples in the chao1 and ace index were significantly different from each other (Figure 3A,B). All of the indices previously mentioned demonstrated that *Reticulitermes flaviceps* had the highest levels of diversity and richness of the bacterial community (Figure 3). Significant differences can be observed when comparing alpha diversity indices across various insect orders and seasons (Figure S2).

Among all aphid species, *Buchnera* was the predominant symbiont. Specifically, the top three symbionts in these species were *Buchnera*, *Wolbachia*, and *Klebsiella*. Notably, *Pseudoregma bambucicola* (summer) and *Ceratovacuna keduensis*, both belonging to the aphid species, exhibited *Buchnera*, *Wolbachia*, and *Klebsiella* as their top three symbionts. However, there were differences in symbionts between *Pseudoregma bambucicola* collected in different seasons (Table S2). Plant hopper species, *Purohita taiwanensis*, and *Tropidocephala brunnipennis* had a higher abundance of Unclassified-Rhizobiaceae as their predominant symbiont. Specifically, *Purohita taiwanensis* had Unclassified-Rhizobiaceae > *Buchnera* > *Wolbachia* as its top three symbionts, while *Tropidocephala brunnipennis* had Unclassified-Rhizobiaceae > *Asaia* > *Candidatus*_Vidania. In contrast, *Reticulitermes flaviceps*, a different insect species, had *Treponema* > *Klebsiella* > *Sphingomonas* as its top three symbionts. Notably, *Treponema* was exclusively found in *Reticulitermes flaviceps* and was not detected in other species (Table S3).

### 3.4. Beta Diversity

**Figure 4:**
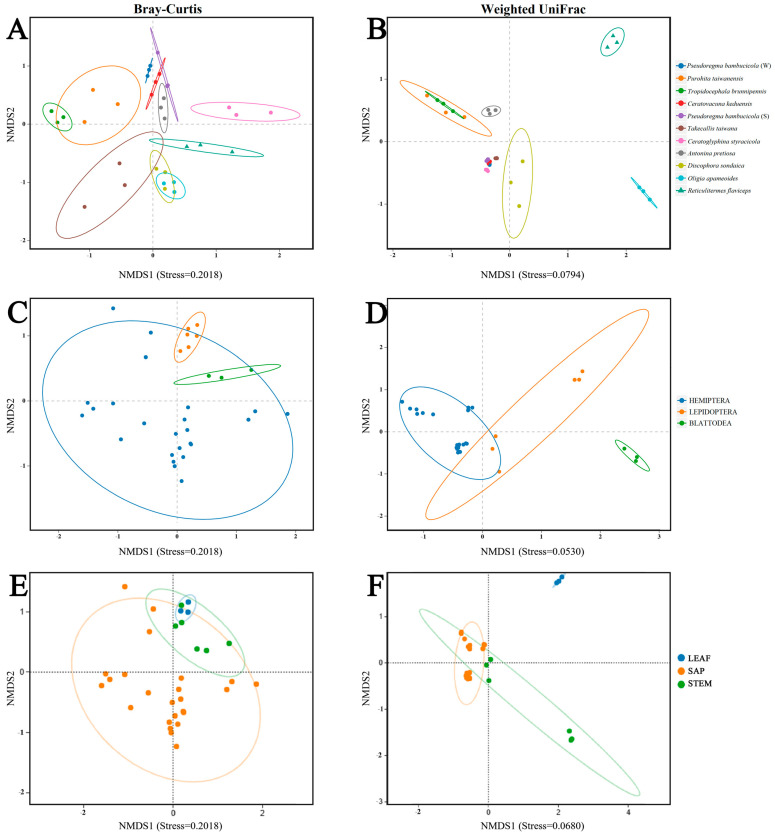
Beta diversity of the bacterial populations is displayed in NMDS plots of *Bambusa* feeding insects. Bray–Curtis distance: (**A**) all insect groups, (**C**) three different insect orders, (**E**) three different diet types and Weighted UniFrac distance (**B**) all insect groups, (**D**) three different insect orders, and (**F**) three different diet types. As shown in the legends, distinct groupings are represented by different colors.

We used Nonmetric Multidimensional Scaling (NMDS) analyses based on the Bray–Curtis and Weighted UniFrac distance metrics to evaluate variations in the compositions of bacterial communities. The stress values for these analyses were 0.02018 and 0.0794, respectively (Figure 4A,B). We also performed NMDS analyses to compare bacterial community differences across three different insect orders (stress = 0.02018, 0.0530) (Figure 4C,D), and three different types of food (stress = 0.02018, 0.0680) (Figure 4E,F). The two most important variables influencing differences between the samples are shown by the ordinate and abscissa axes in the scatter plots. To statistically assess the differences in bacterial populations among various samples, insect orders, food types, and seasons, we used Analysis of Similarities (ANOSIM) and Permutational Multivariate Analysis of Variance (PERMANOVA) (Table 2). These analyses confirmed substantial differences in bacterial populations across all sample categories (Table 2).

**Figure 5:**
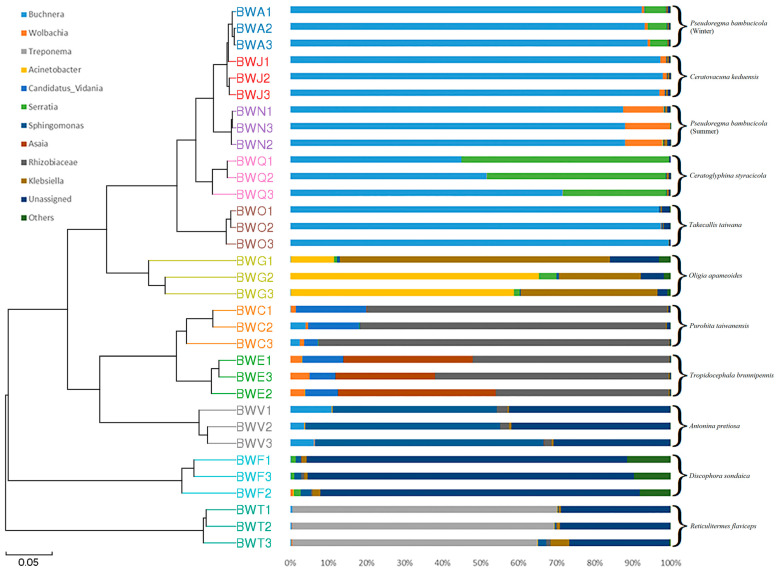
Clustering analysis based on Weighted Unifrac distance algorithm at genus level. See Table 1 for sample information.

The Weighted-Unifrac distance algorithm-based hierarchical clustering tree revealed that all samples were grouped at the genus level. However, there were some notable patterns: aphid species *Pseudoregma bambucicola* (winter), *Ceratovacuna keduensis*, *Pseudoregma bambucicola* (summer), *Ceratoglyphina styracicola*, and *Takecallis taiwana* were closely clustered (Figure 5); this suggests that the flora’s composition was more similar to that of the other insects. However, both species of plant hoppers *Purohita taiwanensis* and *Tropidocephala brunnipennis* clustered with the scale insect *Antonina pretiosa*, which lies between two lepidopteran species *Oligia apameoides* and *Discophora sondaica*. In contrast, *Reticulitermes flaviceps* clustered separately from all other insect species, indicating distinct bacterial community composition (Figure 5).

### 3.5. Functional Prediction of Bacterial Symbionts

The outcomes of the PICRUSt2 analysis are depicted in Figure S3. Several significant differences were found in the *t*-test analyzing the top 10 prevalent functional genes inside metabolic pathways across three different insect orders and dietary preferences.

Blattodea exhibited notably higher relative abundance in “biosynthesis of secondary metabolites”, “biosynthesis of antibiotics”, “biosynthesis of amino acids”, and “purine metabolism” compared to hemipterans and lepidopterans. Conversely, “ABC transporters”, “microbial metabolism in diverse environments”, and “two-component system” displayed significantly higher levels in hemipterans but lower levels in Blattodea. Specifically, “ABC transporters” showed higher levels in hemipterans compared to lepidopterans and were more prevalent in sap feeders compared to shoot feeders (Figure S4A–F).

In terms of diet, leaf eaters exhibited significantly higher relative abundance in “metabolic pathways”, “biosynthesis of secondary metabolites”, “biosynthesis of antibiotics”, and “biosynthesis of amino acids.” Conversely, they showcased less relative abundance in “microbial metabolism in diverse environments”, “ABC transporters”, and “Quorum sensing” compared to shoot and sap feeders (Figure S4D–F).

## 4. Discussion

Symbiotic relationships between insects and their bacterial community play a crucial role in metamorphosis and overall survival. As such, research on the biology of insects needs to take into account the significant influence of these symbionts. There is a notable gap in research that distinguishes changes in symbiotic bacteria among species and their abundance throughout various stages of insect development [4], while some research has documented the diversity of bacteria in insects [43,44]. It is important to recognize that the morphology and physicochemical conditions of insects, as well as their developmental phases, can significantly vary [45]. These variations may impact the unique bacterial ecology specific to each host [46,47]. Understanding these dynamics is vital for a comprehensive understanding of insect biology. Earlier studies, which primarily relied on PCR amplification methods, indicated that interactions with various secondary symbiotic bacteria within a single host insect were uncommon in wild populations [48]. However, modern advancements in HTS have revealed instances of multiple secondary symbiotic bacterial infections within a single insect species [49,50]. Nevertheless, co-infections at the population or individual levels with distinct or identical secondary symbiotic bacteria still remain infrequent [13]. Bamboo-feeding insects have a remarkably rich and diversified bacterial population when compared to other insects, herbivores, and mammals that consume bamboo [51]. This highlights the unique and complex bacterial ecosystems associated with *Bambusa*-feeding insects.

In our study, HTS revealed that the dominant bacterial phyla included *Proteobacteria* (mainly gamma proteobacteria), *Spirochaetota*, *Cyanobacteria Firmicutes*, *Bacteroidota*, *Actinobacteriota*, and *Elusimicrobiota* across all samples. Notably, *Proteobacteria*, *Firmicutes*, and *Bacteroidetes* are commonly found inside various insects and mammals [52]. *Proteobacteria* tend to dominate the microbiota of diverse invertebrates [53], including *H. pensylvanicus* and *A. sanctaecruis* [54], *B. dorsalis* [55], and *H. hampei* [47]. In our study, Hemipteran samples were conquered by *Proteobacteria* and *Firmicutes*, while Lepidopteran insects had three dominant bacterial phyla: *Proteobacteria*, *Cyanobacteria*, and *Firmicutes*. However, previous research has also found *Proteobacteria* and *Firmicutes* as dominant phyla in some Lepidopteran insects like *Bombyx mori*, *Lymantria dispar*, *Manduca sexta*, and *Helicoverpa armigera* [56]. Additionally, *Firmicutes* represent a major component of *B. minax* and *Solenopsis invicta* [57,58]. Moreover, *Spirochaetota* was found to be a major phylum in blattodean insects, consistent with previous studies [59]. Furthermore, *Wolbachia* exhibited varying levels of abundance, with the highest presence observed in *P. bambusicola*, showing significant differences among all samples.

The bacterial communities observed in these herbivorous insects were not solely shaped by convergent evolution with either diet or phylogeny. The bacterial communities were determined by both host diet and phylogeny, which exerted a significant influence on the diversity of bacterial communities [18]. These insects exhibited distinct clustering based on two types of *Bambusa* diets: sap feeding characterized by sucking mouthparts, and leaf and shoot feeding characterized by chewing mouthparts. Our findings revealed significant differences in symbionts between insects feeding on sap compared to those consuming leaves and shoots (Figure 2B and Figure 3B, Table S2). This disparity can be attributed to the structural and chemical complexity of leaves and shoots, which likely involve a more diverse bacterial community in the digestion and absorption process. This observation aligns with previous studies linking higher bacterial diversity to specific dietary preferences [60]. A comparative analysis further demonstrated that both diet and phylogeny significantly influenced the bacterial community composition among *Bambusa*-feeding insects (Figure 5). This outcome is consistent with existing research illustrating the regulation of insect symbionts by factors such as taxonomy and nutrition [18,61]. Previous studies have also emphasized the role of nutrition and phylogeny in shaping bacterial symbiont populations, with the dominant factor varying among different animal species [62]. Traditionally, diet adaptation was considered the primary driver shaping bacterial communities in animals. However, recent studies have challenged this notion, suggesting that host phylogeny and physiology may have a stronger influence on bacterial communities [62]. In polyphagous animals, the interplay between species and nutrition can lead to varying effects on the diversity of bacterial communities. Studies examining the symbiotic bacterial communities of pine processionary moth larvae (*Thaumetopoea pytiocampa*) and fungal farming ants (Tribe: *Attini*) [63] have shown that different diets can significantly influence bacterial diversity within insect species. Additionally, research on longhorn beetles and termites has emphasized the role of diet as the primary factor of bacterial community structure [64]. Interestingly, bacterial communities in xylophagous termites closely resemble those of other xylophagous termites, while detritivorous termites exhibit similarities to detritivorous insects from distantly related orders such as Diptera and Coleoptera [61]. Moreover, this study demonstrated that the composition of the microbiome community did not exhibit differences among closely related host species in terms of phylogeny when the insects were feeding on the same bamboo tissues (Figure 1B, Table S4).

Seasonal fluctuations in the bacterial communities associated with *P. bambusicola* can be observed during summer and winter. Summer-collected *P. bambusicola* exhibited the highest species richness, as indicated by both the chao1 and ace indices (Figure 3 and Figure S2, Table S2), which aligns with previous research [13]. Notably, our results also corroborate their observation that the bacterial symbiont *Serratia* was exclusively detected in *P. bambusicola* collected during the winter season (Figure 1B, Table S3). Currently, *Serratia* is recognized for its role in defending its host insect against various adverse conditions [65]. For example, this symbiont enhances the resistance of aphids to parasitoid wasps and contributes to the ability of aphids to withstand challenging temperatures [66]. On the other hand, *Wolbachia* was detected in *P. bambusicola* collected during the summer season (Figure 1B, Table S3). Typically, *Wolbachia* infection influences the reproduction of its hosts to facilitate its proliferation and transmission. *Wolbachia* infection was rare or nonexistent in *P. nascens* populations in western China, while it was more common in areas farther south that had higher moisture content and milder weather [67]. Similar occurrences were noted in the *C. alternans* and in ants belonging to the genus *Solenopsis* [68,69]. Seasonal variations in bacterial communities have been observed in various insects [15,70,71], further emphasizing the impact of seasonal dynamics on insect-associated bacterial communities.

To forecast the metabolic capacity of bacterial populations, we utilized KEGG ontology analysis, revealing that numerous OTUs exhibited significant metabolic capabilities, irrespective of their association with specific samples (Figure S3). The relative abundance of anticipated gene pathways among the symbiotic bacterial communities does not seem to have varied significantly, according to the PICRUSt2 analysis. This suggests that, based on the predictive functional profiling from the 16S rRNA gene data, there might not be substantial variations in the genetic pathways or functions carried out by these bacterial communities within the studied context. Bacterial phyla like *Proteobacteria* and *Firmicutes* may play a crucial role in nutrition, essential for several physiological processes in the host, and in fixing atmospheric nitrogen, reproduction, and growth [72,73], which were predominantly found across all species (Figure 2A). Plant nutrients play a crucial role in influencing insect fitness, impacting not only the insects themselves but also their symbiotic bacteria, which assist herbivorous insects in efficiently utilizing plant diets that may be limited in essential nitrogen [18,74]. All insects in our study, and especially aphid species, harbor *Buchnera*, which plays a crucial role in providing nutrition. Previous studies reported that *P. bambusicola* loses its ability to feed on *Bambusa*, so their symbiotic bacteria help them to receive nutrition [75]. *Treponema* was only detected in termites (Figure 2B), which help them to degrade cellulose. Recent studies have supported the discovery of cellulolytic bacteria isolated from termites. These findings further corroborate the understanding that these insects possess the ability to break down lignocellulose material [21]. The presence of cellulolytic bacteria within termites and other insects reinforces the idea that these insects rely on symbiotic relationships with these bacterial communities to efficiently degrade and utilize the lignocellulosic compounds present in their diets. This capability is crucial for these insects, allowing them to derive nutrients from plant-based materials that would otherwise be indigestible. This underscores the pivotal role of the insect bacterial community in preserving host physiology. PICRUSt2 research revealed no appreciable differences in the relative abundance of predicted gene pathways in the symbiotic bacterial communities of different insect species. In our observations, we note that the bacterial composition varies across all samples, but they also have some uniqueness as they share 43 of the same OTUs (Table S4), which may help them to feed on the same plant lineage. However, despite their diversity, their core functions appear to be consistent. This commonality in functional attributes may explain why these insects are capable of consuming the same host plant.

## 5. Conclusions

Our study sheds light on the intricate interplay between *Bambusa*-feeding insects and their symbiotic bacterial communities. This study marks the first attempt to compare bacterial communities among insects spanning three distinct orders and diverse feeding habitats, all within the same *Bambusa* species. Our findings underscore the significant variations in symbiotic bacteria presence, abundance, and composition across the studied samples. They emphasize the multifaceted factors influencing these communities, from dietary choices and seasonal variations to host phylogeny. Insect species feeding on the same plant lineage can have different bacterial communities but with congruent functional abilities. These insights contribute to our understanding of the diverse and dynamic world of insect–bacterial interactions, with potential implications for insect biology, ecology, and beyond.

## Figures and Tables

**Table 1:** Detailed sample information used in this study.

Sr. No	Collection Date	Sample ID	Host Plant	Insect Specie	Insect Order	Feeding	Sample Name in Figures
1	1 November 2022	BWA1	*Bambusa* sp.	*Pseudoregma bambucicola* (Winter)	Hemiptera	Sap	
2		BWA2					PbW.Sa.H
3		BWA3					
4	4 May 2023	BWC1	*Bambusa* sp.	*Purohita taiwanensis*		Sap	
5		BWC2					Pt.Sa.H
6		BWC3					
7	6 May 2023	BWE1	*Bambusa* sp.	*Tropidocephala brunnipennis*		Sap	
8		BWE2					Tb.Sa.H
9		BWE3					
10	8 May 2023	BWO1	*Bambusa* sp.	*Takecallis taiwana*		Sap	
11		BWO2					Tt.Sa.H
12		BWO3					
13	9 May 2023	BWJ1	*Bambusa* sp.	*Ceratovacuna keduensis*		Sap	
14		BWJ2					Ck.Sa.H
15		BWJ3					
16	9 May 2023	BWN1	*Bambusa* sp.	*Pseudoregma bambucicola* (Summer)		Sap	
17		BWN2					PbS.Sa.H
18		BWN3					
19	9 May 2023	BWQ1	*Bambusa* sp.	*Ceratoglyphina styracicola*		Sap	
20		BWQ2					Ct.Sa.H
21		BWQ3					
22	4 June 2023	BWV1	*Bambusa* sp.	*Antonina pretiosa*		Sap	
23		BWV2					Ap.Sa.H
24		BWV3					
25	11 June 2023	BWF1	*Bambusa* sp.	*Discophora sondaica*	Lepidoptera	Leaf	
26		BWF2					Ds.Lf.L
27		BWF3					
28	2 May 2023	BWG1	*Bambusa* sp.	*Oligia apameoides*		Stem	
29		BWG2					Oa.St.L
30		BWG3					
31	8 May 2023	BWT1	*Bambusa* sp.	*Reticulitermes flaviceps*	Blattodea	Stem	
32		BWT2					Rf.St.B
33		BWT3					

**Table 2:** Results of ANOSIM and PERMANOVA based on Bray–Curtis and Weighted Unifrac distances.

Beta Diversity Distance	Bacterial Community	ANOSIM (*R, p*)	PERMANOVA (*R*^2^*, p*)
Bray–Curtis	All Samples	0.935, *0.001*	0.936, *0.001*
	Insect Orders	0.223, *0.003*	0.235, *0.001*
	Type of food (Sap, Leaf, Stem)	0.189, *0.005*	0.217, *0.001*
	Season (Summer, Winter)	0.481, *0.01*	0.240, *0.01*
Weighted Unifrac	All Samples	0.888, *0.001*	0.970, *0.001*
	Insect Orders	0.721, *0.001*	0.509, *0.001*
	Type of food (Sap, Leaf, Stem)	0.718, *0.001*	0.483, *0.001*
	Season (Summer, Winter)	0.333, 0.02	0.174, 0.09

Italicize statistically significant *p* values (*p* < 0.01); see Table 1 for sample information.

## Data Availability

The raw sequencing data are deposited in the NCBI Sequence Read Archive (SRA) database under accession number PRJNA1045400.

## Supplementary Materials

The following supporting information can be downloaded at https://www.mdpi.com/article/10.3390/insects15030187/s1, Table S1: The Illumina HiSeq sequencing results of bacterial 16S rRNA gene; Table S2: Detailed information of symbionts diversity indices of all samples; Table S3: Percentage of relative abundance of top ten major symbiotic bacteria in all samples; Table S4: Percentage of relative abundance and their number of reads of commonly shared unique symbiotic bacteria in all samples; Figure S1: Shannon rarefaction curves for all samples; Figure S2: Comparison of the bacterial communities’ alpha diversity. Between different insect orders, between different seasons; Figure S3: Relative abundance of predicted genes in top 30 abundant pathways identified by the PICRUSt2 analysis; Figure S4: *t*-test results of top ten abundant pathways for the comparison of three different insect orders and diet.
