# Supplementary material for: Symbiotic Bacterial Communities of Insects Feeding on the Same Plant Lineage: Distinct Composition but Congruent Function
Source: Insects. 2024 Mar 12;15(3):187. doi: 10.3390/insects15030187 (PMC10970990; doi:10.3390/insects15030187)
Supplement: Supplementary file 1 [file insects-15-00187-s001.zip › insects-2790976-supplementary.pdf]

Supplementary Materials

Symbiotic Bacterial Communities of Insects Feeding on the Same Plant Lineage: Distinct Composition but Congruent Function

Waleed Afzal Naveed , Qian Liu , Congcong Lu and Xiaolei Huang *

Key Laboratory of Ecological Pest Control for Fujian and Taiwan Crops, College of Plant Protection, Fujian Agriculture and Forestry University, Fuzhou 350002, China; waleedafzal75@gmail.com (W.A.N.); liuqian9502@163.com (Q.L.); lcchuaer613@126.com (C.L.)

* Correspondence: huangxl@fafu.edu.cn; Tel.: +86-0591-83705205

Table S1. The Illumina HiSeq sequencing results of bacterial 16S rRNA gene. Raw tags = number of 16S rDNA sequences after merged the PE reads, Clean tags = number of 16S rDNA sequences after further quality filtering the Raw tags, Reads = number of 16S rDNA sequences after discard the OTUs with a number of sequences < 0.005% of the total number of sequences. See Table 1 for detailed sample information.

Sample ID	Species Name	Raw Tags	Clean Tags	Reads
BWA1	*Pseudoregma bambucicola* (Winter)	79717	75425	74875
BWA2		80141	75521	74932
BWA3		79993	75575	75113
BWC1	*Purohita taiwanensis*	80153	75863	75571
BWC2		79925	75441	75067
BWC3		79880	75671	75454
BWE1	*Tropidocephala brunnipennis*	79675	75377	74788
BWE2		80036	75789	75171
BWE3		79893	75754	74591
BWF1	*Discophora sondaica*	49054	46620	45956
BWF2		42227	40081	39349
BWF3		50268	47591	46325
BWG1	*Oligia apameoides*	53964	51039	50236
BWG2		46457	44084	41885
BWG3		80037	76295	67808
BWJ1	*Ceratovacuna keduensis*	79844	75241	74241
BWJ2		80167	75560	74555
BWJ3		80413	75753	74727
BWN1	*Pseudoregma bambucicola* (Summer)	80130	75704	74962
BWN2		79950	75944	75077
BWN3		80225	75699	74845
BWO1	*Takecallis taiwana*	40894	38612	38306
BWO2		55224	52353	52087
BWO3		82586	78144	77892
BWQ1	*Ceratoglyphina styracicola*	80086	76126	75873
BWQ2		80090	75533	75037
BWQ3		80121	75565	75006
BWT1	*Reticulitermes flaviceps*	80041	75876	75319
BWT2		80054	75791	75106
BWT3		79802	75494	74241
BWV1	*Antonina pretiosa*	79994	75525	74643
BWV2		79909	75406	74317
BWV3		79985	75727	74134

Table S2. Detailed information of symbionts diversity indices of all samples. OTUs, number of operational taxonomic units at a 0.03 cut-off; ACE and Chao1, estimates of species richness; Simpson and Shannon, estimates of diversity; Coverage, the probability of species being measured in the sample. See Table 1 for detailed sample information.

Sample ID	Species Name	OTUs	ACE	Chao1	Simpson	Shannon	Coverage
BWA1	*Pseudoregma bambucicola* (Winter)	143	174.42	180.40	0.42	1.37	0.95
BWA2		141	189.92	194.45	0.19	0.76	0.93
BWA3		145	184.12	196.75	0.18	0.72	0.94
BWC1	*Purohita taiwanensis*	169	434.50	418.00	0.23	0.90	0.99
BWC2		177	224.50	220.24	0.44	1.57	0.94
BWC3		78	89.73	97.43	0.21	0.80	0.98
BWE1	*Tropidocephala brunnipennis*	110	201.32	157.05	0.63	1.79	0.94
BWE2		115	172.92	172.50	0.60	1.79	0.94
BWE3		123	230.76	178.43	0.65	1.88	0.93
BWF1	*Discophora sondaica*	165	294.82	231.00	0.67	3.60	0.92
BWF2		127	148.66	136.33	0.80	4.55	0.98
BWF3		170	310.44	213.50	0.72	4.04	0.93
BWG1	*Oligia apameoides*	145	147.03	146.50	0.72	3.45	0.99
BWG2		109	116.50	116.20	0.81	3.55	0.98
BWG3		173	190.24	198.67	0.81	3.48	0.96
BWJ1	*Ceratovacuna keduensis*	176	221.87	225.00	0.16	0.68	0.93
BWJ2		184	236.87	239.19	0.26	0.84	0.92
BWJ3		202	255.74	279.00	0.24	0.95	0.92
BWN1	*Pseudoregma bambucicola* (Summer)	273	367.63	356.44	0.40	1.39	0.98
BWN2		71	89.08	96.30	0.40	1.16	0.97
BWN3		258	369.04	397.79	0.42	1.43	0.98
BWO1	*Takecallis taiwana*	64	74.86	69.00	0.12	0.64	0.98
BWO2		104	121.81	119.11	0.13	0.69	0.97
BWO3		58	63.83	63.14	0.02	0.13	0.99
BWQ1	*Ceratoglyphina styracicola*	76	84.45	89.00	0.51	1.13	0.98
BWQ2		226	343.71	343.00	0.52	1.30	0.98
BWQ3		226	310.91	321.29	0.43	1.16	0.99
BWT1	*Reticulitermes flaviceps*	372	420.09	418.41	0.95	6.02	0.92
BWT2		396	430.41	443.30	0.94	6.17	0.93
BWT3		517	544.25	550.78	0.96	6.64	0.92
BWV1	*Antonina pretiosa*	303	331.59	349.87	0.95	5.85	0.94
BWV2		338	455.91	446.50	0.92	5.58	0.91
BWV3		326	383.98	425.75	0.90	5.30	0.92

Table S3. Percentage of relative abundance of top ten major symbiotic bacteria in all samples.

Sample ID	Insect Specie	Insect Order	*Buchnera*	Rhizobiaceae	*Wolbachia*	*Treponema*	*Acinetobacter*	*Candidatus_Vidania*	*Serratia*	*Sphingomonas*	Asaia	Klebsiella	Unassign	Others
BWA1	*Pseudoregma bambucicola* (Winter)	Hemiptera	92.40	0.23	0.64	0.02	0.01	0.05	5.54	0.09	0.00	0.29	0.62	0.11
BWA2			93.17	0.16	0.74	0.00	0.01	0.01	5.09	0.06	0.00	0.20	0.47	0.10
BWA3			93.98	0.15	0.55	0.00	0.01	0.02	4.52	0.05	0.00	0.22	0.43	0.07
BWN1	*Pseudoregma bambucicola* (Summer)		87.48	0.12	10.62	0.08	0.07	0.00	0.07	0.17	0.00	0.54	0.77	0.07
BWN3			88.07	0.19	9.68	0.09	0.12	0.00	0.10	0.20	0.02	0.69	0.79	0.05
BWN2			88.02	0.02	11.77	0.00	0.02	0.00	0.01	0.00	0.00	0.02	0.12	0.01
BWJ1	*Ceratovacuna keduensis*		97.34	0.05	1.50	0.00	0.01	0.00	0.02	0.12	0.00	0.45	0.43	0.07
BWJ2			97.94	0.09	0.98	0.02	0.02	0.00	0.00	0.12	0.00	0.41	0.37	0.05
BWJ3			97.07	0.12	1.39	0.01	0.04	0.00	0.00	0.17	0.00	0.46	0.67	0.06
BWO1	*Takecallis taiwana*		96.87	0.09	0.00	0.00	0.06	0.00	0.00	0.36	0.00	0.33	2.03	0.26
BWO2			97.48	0.12	0.03	0.00	0.06	0.00	0.00	0.37	0.05	0.16	1.57	0.16
BWO3			99.45	0.02	0.01	0.00	0.01	0.00	0.00	0.02	0.00	0.06	0.43	0.00
BWQ1	*Ceratoglyphina styracicola*		45.02	0.01	0.00	0.00	0.01	0.00	54.53	0.03	0.00	0.05	0.36	0.00
BWQ2			51.56	0.11	0.02	0.08	0.02	0.00	47.03	0.11	0.00	0.50	0.53	0.04
BWQ3			71.55	0.08	0.02	0.05	0.00	0.00	27.20	0.13	0.00	0.45	0.44	0.07
BWC1	*Purohita taiwanensis*		0.14	79.04	1.30	0.00	0.05	18.51	0.01	0.04	0.01	0.29	0.54	0.06
BWC2			4.03	80.30	0.65	0.03	0.01	13.35	0.16	0.15	0.00	0.28	0.97	0.09
BWC3			2.49	92.28	1.19	0.00	0.01	3.57	0.10	0.04	0.00	0.03	0.23	0.06
BWE1	*Tropidocephala brunnipennis*		0.00	51.64	3.04	0.00	0.01	10.78	0.00	0.04	34.13	0.16	0.17	0.03
BWE2			0.02	61.48	4.96	0.00	0.05	6.74	0.00	0.04	26.18	0.26	0.23	0.03
BWE3			0.00	45.45	3.81	0.00	0.02	8.60	0.00	0.06	41.56	0.28	0.18	0.03

BWV1	*Antonina pretiosa*		10.80	2.84	0.16	0.03	0.04	0.01	0.00	43.23	0.04	0.31	42.45	0.08
BWV2			3.59	2.39	0.10	0.05	0.16	0.01	0.00	51.20	0.03	0.54	41.83	0.08
BWV3			6.26	2.01	0.11	0.05	0.08	0.01	0.00	60.15	0.04	0.55	30.65	0.09
BWG1	*Oligia apameoides*	Lepidoptera	0.10	0.02	0.00	0.00	11.43	0.00	0.73	0.78	0.00	71.03	12.89	3.03
BWG2			0.02	0.09	0.00	0.00	65.37	0.00	4.64	0.54	0.00	21.49	6.04	1.81
BWG3			0.13	0.02	0.01	0.00	58.66	0.00	1.58	0.23	0.00	35.89	2.55	0.94
BWF1	*Discophora sondaica*		0.09	0.02	0.08	0.00	0.02	0.00	1.22	1.38	0.19	1.24	84.27	11.48
BWF2			0.11	0.69	0.01	0.00	0.01	0.00	0.95	1.76	0.07	0.94	85.77	9.69
BWF3			0.08	0.41	0.57	0.00	0.29	0.00	1.82	2.68	0.00	2.09	83.93	8.14
BWT1	*Reticulitermes flaviceps*	Blattodea	0.56	0.16	0.01	69.55	0.08	0.00	0.07	0.16	0.00	0.62	28.71	0.09
BWT2			0.46	0.20	0.04	68.81	0.10	0.00	0.08	0.35	0.00	0.83	28.95	0.17
BWT3			0.26	1.15	0.29	64.35	0.22	0.00	0.00	2.18	0.00	4.83	26.30	0.40

Table S4. Percentage of relative abundance and their number of reads of commonly shared unique symbiotic bacteria in all samples.

Sr. No.	Order	Family	Genus	OTU_id	Hemiptera								Lepidoptera		Blattodea
					Pseudoregma bambucicola (Winter)	*Pseudoregma bambucicola* (Summer)	*Purohita taiwanensis*	*Tropidocephala brunnipennis*	*Ceratovacuna keduensis*	*Takecallis taiwana*	*Ceratoglyphina styracicola*	*Antonina pretiosa*	*Oligia apameoides*	*Discophora sondaica*	*Reticulitermes flaviceps*
1	Acidobacteriales	Acidobacteriaceae	Acidobacterium	OTU2703	10	5	15	3	5	9 (0.29%)	14 (0.69%)	2	107 (0.14%)	68	5
2	Burkholderiales	Alcaligenaceae	Achromobacter	OTU155	8	8	16	1	15	34 (1.09%)	13 (0.64%)	9	301 (0.39%)	1	13 (0.21%)
3		Nitrosomonadaceae	MND1	OTU1113	5	2	8	1	7	26 (0.83%)	2	7	1	66	16 (0.25%)
4		Comamonadaceae	Ramlibacter	OTU1560	3	6	3	6	21	6 (0.19%)	4	9	7	24	46 (0.73%)
5		TRA3 20	-	OTU473	1	2	1	2	6	13	11	1	112 (0.14%)	61	3
6	Caulobacterales	Caulobacteraceae	Phenylobacterium	OTU660	1	4	5	3	25	28 (0.90%)	4	621 (0.95%)	55	1	17 (0.27%)
7	Chloroflexi	-	-	OTU270	1	1	15	2	2	1	1	4	181 (0.23%)	87	2
8	Clostridia UCG_014	-	-	OTU1426	5	8	14	2	8	24 (0.77%)	6	4	2	11	47 (0.74%)

9	Clostridiales	Clostridiaceae	Clostridium_sensu_stricto	OTU140	1	18	2	2	13	108 (3.46%)	3	11	382 (0.49%)	148	19
10	Cyanobacteriales	-	-	OTU66	648 (0.39%)	3	334 (0.18%)	145 (0.13%)	103	22 (0.71%)	1	12	1	3	5
11		-	-	OTU6	3	3	64	3	22	74 (2.37%)	27 (1.33%)	28	60819 (77.98%)	2497 (0.40%)	5
12	Enterobacterales	Morganellaceae	Buchnera	OTU1	197773 (95.61%)	165978 (86.63%)	4545 (2.51%)	18	190760 (98.46%)	1149 (36.83%)	471 (23.25%)	9680 (14.79%)	43	73	420 (6.63%)
13		Enterococcaceae	Escherichia_Shigella	OTU47	28	81	115	33	117	31 (0.99%)	31 (1.53%)	47	965 (1.24%)	340	87 (1.37%)
14		Enterobacteriaceae	klebsiella	OTU68	274 (0.13%)	45	173 (0.10%)	35	372 (0.19%)	24 (0.77%)	38 (1.88%)	79 (0.12%)	189 (0.24%)	6647 (1.08%)	125 (1.97%)
15				OTU12	25	17	46	6	30	16 (0.51%)	19 (0.94%)	7	164 (0.21%)	34839 (5.65%)	55 (0.87%)
16				OTU39	195	403 (0.21%)	183	452 (0.40%)	463 (0.24%)	198 (6.35%)	364 (17.97%)	413 (0.63%)	1001 (1.28%)	932(0.15%)	1708 (26.94%)
17		Pectobacteriaceae	Pectobacterium	OTU23	6342 (3.07%)	23605 (12.32%)	43	5	4	116 (3.72%)	30 (1.48%)	584 (0.89%)	102 (0.13%)	6	35 (0.55%)
18	Frankiales	-	-	OTU567	6	14	8	4	6	9 (0.29%)	11 (0.54%)	408 (0.62%)	1	96	77 (1.21%)

19	Gemmatimonadales	Gemmatimonadaceae	Gemmatirosa	OTU1847	7	7	5	2	7	80 (2.56%)	2	5	9	53	5
20	Holophagae, Subgroup 7	-	-	OTU194	1	6	1	1	1	11 (0.35%)	4 (0.20%)	1	412 (0.53%)	48	14 (0.22%)
21	Lactobacillales	Enterococcaceae	Enterococcus	OTU29	548 (0.26%)	437 (0.23%)	858 (0.47%)	270 (0.24%)	642 (0.33%)	63 (2.02%)	196 (9.67%)	853 (1.30%)	111 (0.14%)	438	907 (14.31%)
22		Lactobacillaceae	Lactiplantibacillus	OTU170	3	30	99	3	97	40 (1.28%)	30 (1.48%)	1	501 (0.64%)	301	24 (0.38%)
23			Lactobacillus	OTU127	22	16	96	9	29	120(3.85%)	24 (1.18%)	2	337 (0.43%)	671 (0.11%)	139 (2.19%)
24				OTU189	9	20	108	1	63	95 (3.04%)	33 (1.63%)	3	126 (0.16%)	138	108 (1.70%)
25			Ligilactobacillus	OTU146	2	17	89	3	61	4 (0.13%)	22 (1.09%)	6	21	625 (0.10%)	53 (0.84%)
26		Streptococcaceae	Streptococcus	OTU178	7	27	3	9	24	5 (0.16%)	3 (0.15%)	2	232 (0.30%)	20	31 (0.49%)
27	Micrococcales	Micrococcaceae	Arthrobacter	OTU83	55	95	62	42	195	41 (1.31%)	88 (4.34%)	168 (0.26%)	201 (0.26%)	166	382 (6.03%)
28	Peptostreptococcales	Peptostreptococcaceae	Romboutsia	OTU177	3	24	29	14	14	18 (0.58%)	30 (1.48%)	33	282 (0.36%)	255	18 (0.28%)
29	Pseudomonadales	Pseudomonadaceae	Pseudomonas	OTU22	81	10	37	490 (0.44%)	93	33 (1.06%)	24 (1.18%)	27	152 (0.19%)	8003 (1.30%)	62 (0.98%)

30				OTU49	32	5	33	4	14	1	7 (0.35%)	10	7	2398 (0.39%)	7 (0.11%)
31				OTU3757	20	19	16	15	11	16 (0.51%)	7 (0.35%)	36	19	8	36 (0.57%)
32	Rhizobiales	Rhizobiaceae	-	OTU2	304 (0.15%)	27	173730 (95.89%)	110656 (98.41%)	21	5 (0.16%)	1	126 (0.19%)	16	17	27 (0.43%)
33			-	OTU250	10	25	11	18	27	34 (1.09%)	9 (0.44%)	178 (0.27%)	132 (0.17%)	1	99 (1.56%)
34			-	OTU461	3	9	2	1	4	14 (0.45%)	11 (0.54%)	7	88 (0.11%)	19	7 (0.11%)
35		Xanthobacteraceae	-	OTU41	30	20	27	12	51	27 (0.87%)	52 (2.57%)	2378 (3.63%)	49	166	63 (0.99%)
36			-	OTU2412	7	2	6	4	8	57 (1.83%)	3 (0.15%)	10	6	4	21 (0.33%)
37	Sphingomonadales	Sphingomonadaceae	Novosphingobium	OTU561	20	11	5	3	19	71 (2.28%)	21 (1.04%)	308 (0.47%)	2	26	59 (0.93%)
38			Sphingomonas	OTU21	48	77	44	51	79	126 (4.04%)	72 (3.55%)	48554 (74.17%)	856 (1.10%)	251	463 (7.30%)
39				OTU2681	29	43	62	18	57	12 (0.38%)	41 (2.02%)	71 (0.11%)	13	50	133 (2.10%)

40				OTU248	4	7	2	3	10	8 (0.26%)	10 (0.49%)	301 (0.46%)	183 (0.23%)	1	13 (0.21%)
41	Staphylococcales	Staphylococcaceae	Staphylococcus	OTU136	25	48	90	3	42	64 (2.05%)	7 (0.35%)	43	576 (0.74%)	332	24 (0.38%)
42	unclassified_Bacteria			OTU18	167	84	124	58	113	166 (5.32%)	60 (2.96%)	118 (0.18%)	9102 (11.67%)	1616 (0.26%)	244 (3.85%)
43				OTU2571	88	321 (0.17%)	42	31	92	121 (3.88%)	219 (10.81%)	297 (0.45%)	123 (11.67%)	153	715 (11.28%)

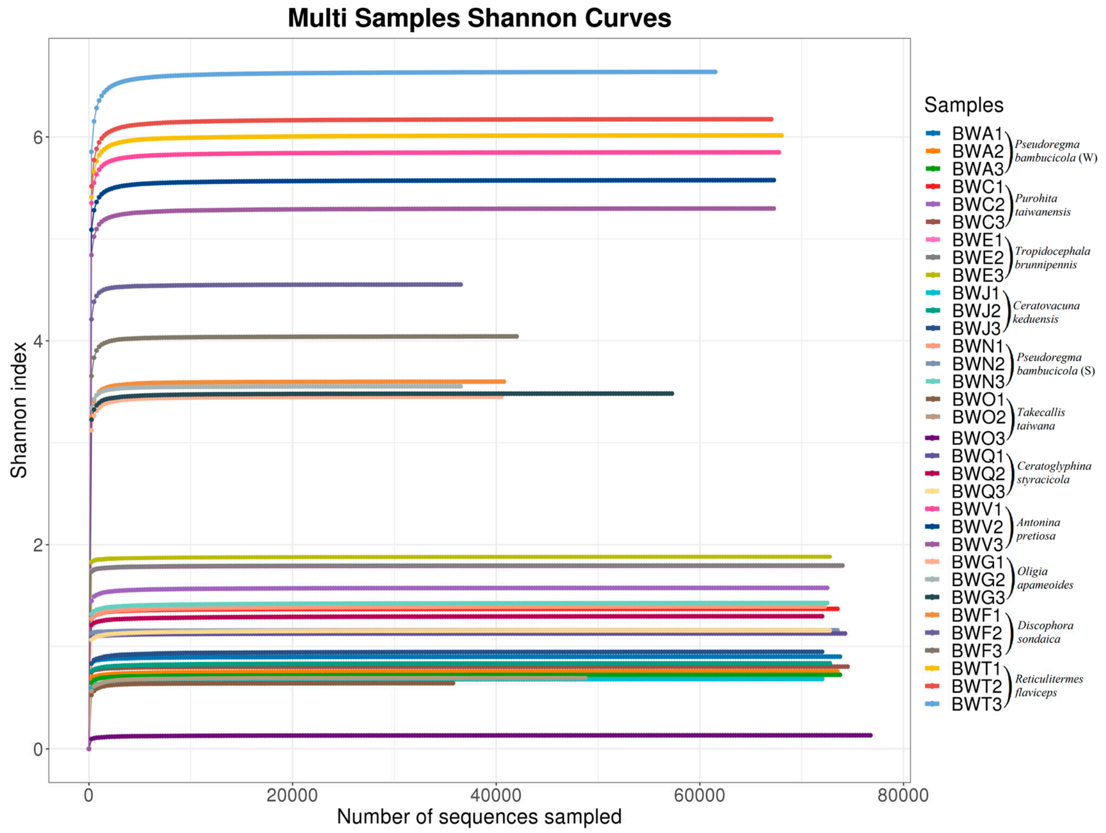

Figure S1. Shannon rarefaction curves for all samples.

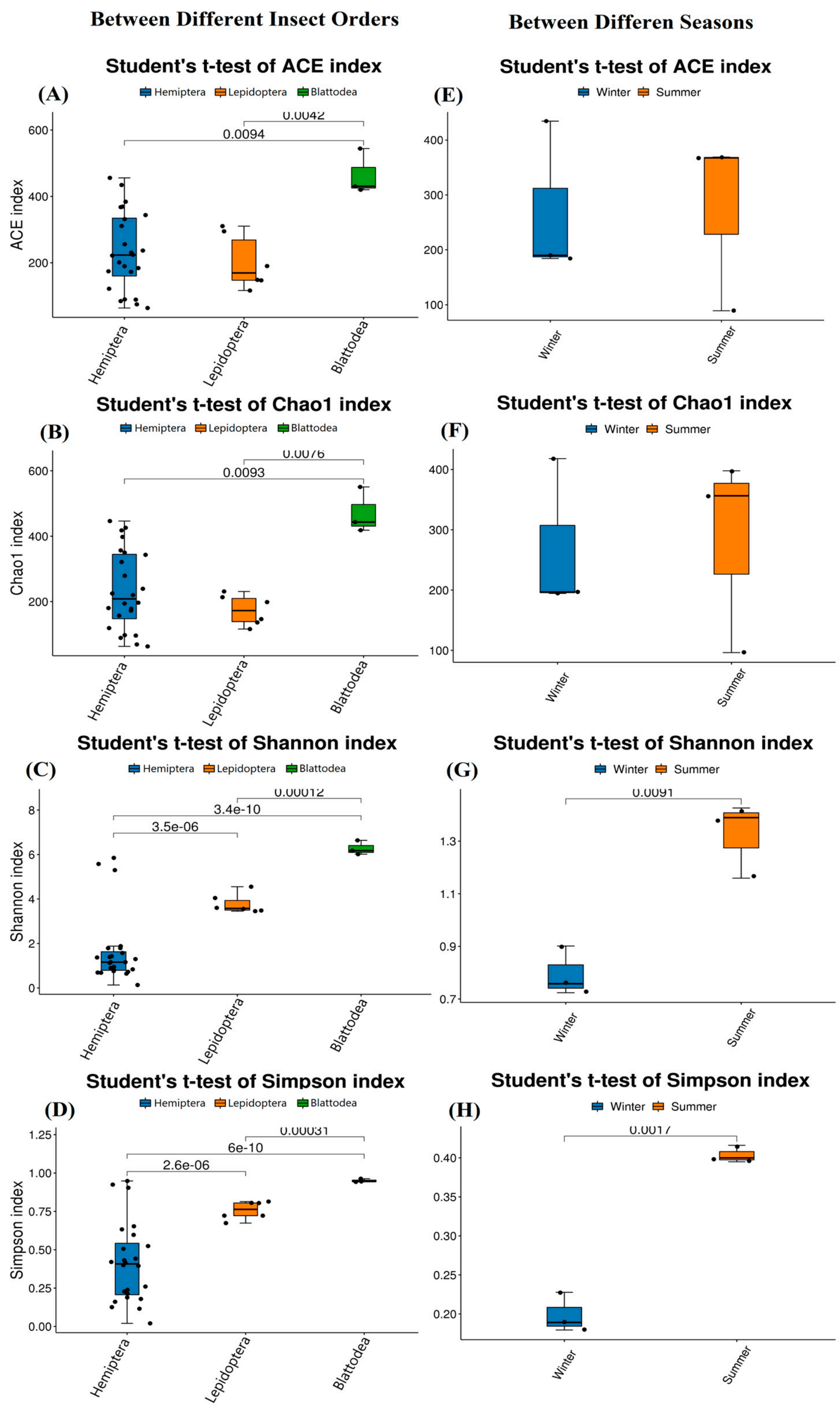

Figure S2. Comparison of the bacterial communities' alpha diversity. Between different insect orders; (A) ACE index, (B) Chao1 index, (C) Shannon index, (D) Simpson index, Between different seasons; (E) ACE index, (F) Chao1 index, (G) Shannon index, (H) Simpson index. Note: X-axis: Group name; Y-axis: Alpha diversity indices. The line inside the boxplots represent medians, the dots in the center are the means and the whiskered bars are maximal and minimal values. The value indicates P-value calculated by t-test (If the P value > 0.05).

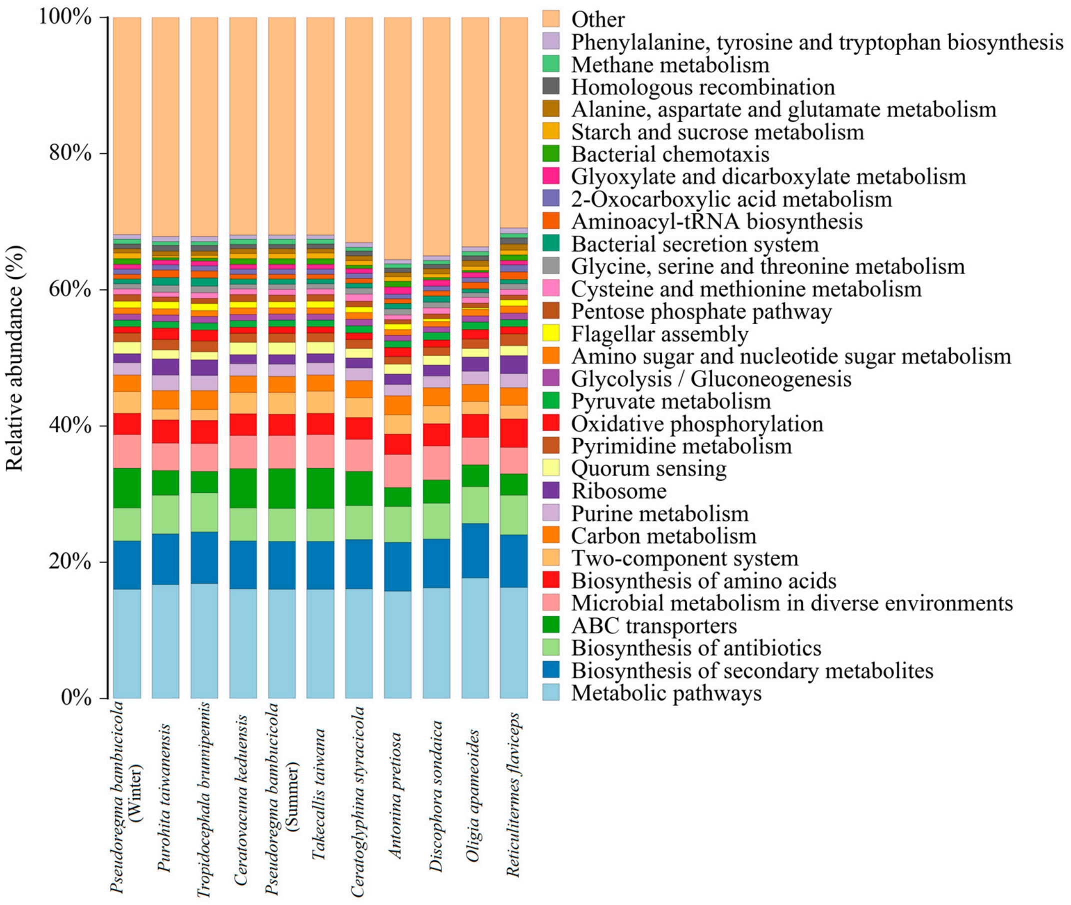

Figure S3. Relative abundance of predicted genes in top 30 abundant pathways identified in the symbiotic bacterial communities of *Bambusa* feeding insects by the PICRUSt2 analysis. The pathways are presented according to KEGGs.

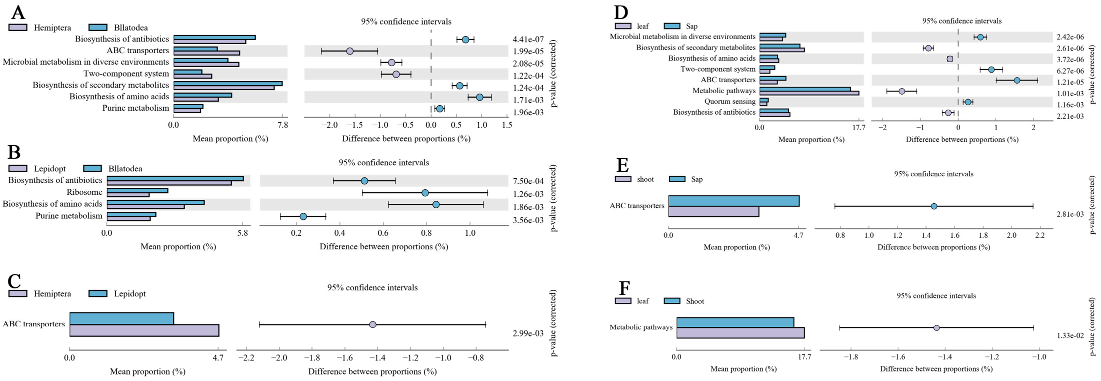

Figure S4. T-test results of top ten abundant pathways for the comparison of three different insect orders and diet. Only shown the pathways that with significant differences in relative abundance. See Table 1 for detailed sample information
